# Study of the nitric oxide system in the rat cerebellum during aging

**DOI:** 10.1186/1471-2202-11-78

**Published:** 2010-06-24

**Authors:** Santos Blanco, Francisco J Molina, Lourdes Castro, Maria L Del Moral, Raquel Hernandez, Ana Jimenez, Alma Rus, Esther Martinez-Lara, Eva Siles, Maria A Peinado

**Affiliations:** 1 Department of Experimental Biology, University of Jaen. Campus Las Lagunillas s/n, 23071, Jaén, Spain

## Abstract

**Background:**

The cerebellum is the neural structure with the highest levels of nitric oxide, a neurotransmitter that has been proposed to play a key role in the brain aging, although knowledge concerning its contribution to cerebellar senescence is still unclear, due mainly to absence of integrative studies that jointly evaluate the main factors involved in its cell production and function. Consequently, in the present study, we investigate the expression, location, and activity of nitric oxide synthase isoenzymes; the protein nitration; and the production of nitric oxide in the cerebellum of adult and old rats.

**Results:**

Our results show no variation in the expression of nitric oxide synthase isoforms with aging, although, we have detected some changes in the cellular distribution pattern of the inducible isoform particularly in the cerebellar nuclei. There is also an increase in nitric oxide synthase activity, as well as greater protein-nitration levels, and maintenance of nitrogen oxides (NOx) levels in the senescent cerebellum.

**Conclusions:**

The nitric oxide/nitric oxide syntahses system suffers from a number of changes, mainly in the inducible nitric oxide synthase distribution and in overall nitric oxide synthases activity in the senescent cerebellum, which result in an increase of the protein nitration. These changes might be related to the oxidative damage detected with aging in the cerebellum.

## Background

The cerebellum is the neural structure that produces the highest levels of nitric oxide (NO) within the central nervous system (CNS) [1]. This high levels of NO may make cerebellum more susceptible to oxidative and nitrosative stress, particularly in the senescence [2]. However, only a few preliminary studies from our research group [3,4] and others [5] have tried to set a basic approach concerning the involvement of NO in the senescent cerebellum.

**Figure 1:**
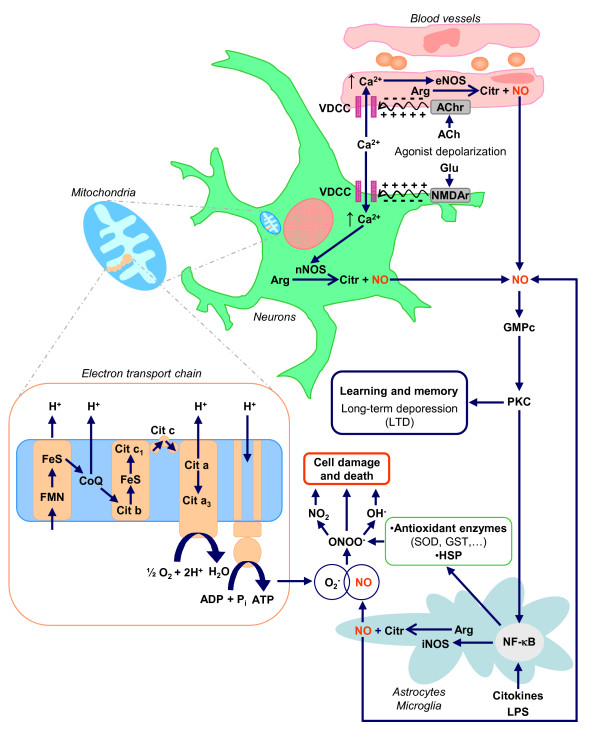
**Schematic model summarizing the possible role of the three NOS isoforms in the aging cerebellum**. The NO produced by eNOS and nNOS facilitates the learning and memory processes in the cerebellum through the NO-GMPc-PKC pathway. During aging, cerebellar cells accumulate reactive oxygen species which in turn can react with the iNOS-derived NO and form peroxynitrite that causes cell damage and death. ACH, acetylcholine; Glu, glutamate; VDCC: voltage-dependent calcium channels.

According to Harman's theory of free radicals [6], aging results from the harmful effects of free radicals produced during cell metabolism, reactive oxygen species (ROS) being the main agents responsible for this process. More recently, reactive nitrogen species (RNS), such as NO, and in particular its derivatives, have been associated with the oxidative damage that occurs during aging [7,8]. In this light, and given that the gaseous neurotransmitter NO plays a major role in cerebellar functionality [9-11], the study of the NO-producing system in the cerebellum is essential to understand how aging affects cerebellar functionality. Moreover, according to McCann's hypothesis [7] about the role of NO in the aging brain, hyperactivity of the glutamatergic pathway could boost activity of certain calcium-dependent enzymes, such as the nitric oxide synthase enzymes (NOS). In mammals, three different NOS isoforms have been identified: neuronal NOS (nNOS), endothelial NOS (eNOS) and inducible NOS (iNOS). Due to its high reactivity, NO can react with the superoxide anion (O_2_^-^) to generate the free radical peroxynitrite (ONOO^-^) (Figure 1), which in turn can react with the phenolic ring of tyrosine residues of proteins to form 3-NL-tyrosine (nitrotyrosine, n-Tyr). The formation of n-Tyr can alter the structure, and therefore the function of proteins, so that this compound is often used as a marker of cumulative exposure to NO [12]. The increase in the formation of n-Tyr has been correlated with a wide variety of neurodegenerative diseases (Alzheimer's, Parkinson's, amyotrophic lateral sclerosis) as well as with atherosclerosis, with hypoxia and ischemia, and even with aging [13-15]. However, NO also plays key roles at the physiological level; thus, it acts as an inter- and intracellular messenger, triggering protective functions in the cerebellum which include vasodilatation (Figure 1) and participation in the synaptic plasticity mechanism of long-term depression (LTD) (Figure 1) [9-11,16,17], which is essential in motor learning and regulatory functions. In short, it can be established that NO performs a dual role as protective and toxic molecule, depending mainly on its production, and consequently on the concentration reached within the tissue.

Although many studies have pointed to the gaseous neuromodulator nitric oxide (NO) as a key molecule in the whole brain-aging process [18], knowledge about its involvement in the senescent cerebellum is still unclear, due to absence of studies that analyse both the NOS expression and location within the cerebellum during aging. In this sense it is necessary to carry out a comprehensive study that covers all these as yet unexplained facts.

Considering all these antecedents, we hypothesize that aging could develop an overall key role in the modulation of the NO/NOS system in the cerebellum. Consequently, we analysed the response of the NO/NOS system during aging in the cerebellum of the rat. For this, we examined the expression, location and activity of the three nitric oxide synthase isoforms (nNOS, eNOS and iNOS) on mature adults (4 months old) and aged rats (26 months old). The study was completed with the analysis of the nitrotyrosine expression and location in the different experimental groups, and the measurement of nitrogen oxides (NOx) levels.

## Results

### 2.1. NOS isoform expression and location

**Figure 2:**
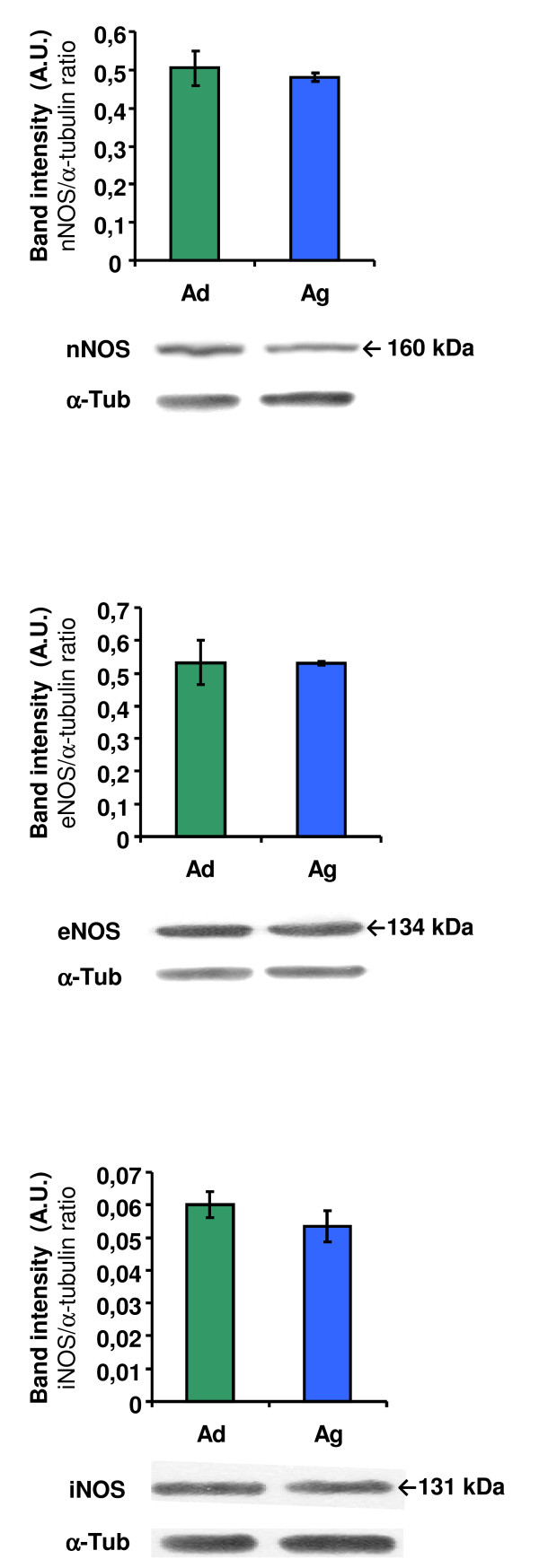
**Western-blot analysis of nNOS, eNOS and iNOS expression in the rat cerebellum**. Top panels: densitometric quantification of the NOS isoforms in the cerebellum of adult and aged rats (Ad, Ag). Bottom panels: representative autoradiographies of the corresponding nNOS, eNOS and iNOS bands; α-tubulin immunodetection was also included as a protein-loading control. Results are average values of 5 experimental animals in each group and are expressed as ratio of band intensities of the NOS isoform and its corresponding α-tubulin. Data are the means (± S.D.) of five determinations. A.U.: arbitrary units. No statistical differences detected.

To elucidate the age-related expression patterns of the NOS isoforms in the cerebellum, we performed a western-blot analysis using nNOS, eNOS and iNOS antibodies in rat cerebellar samples of two experimental groups: adult (Ad) and aged (Ag) animals (Figure 2). In all cases, we detected a similar expression pattern; and no statistical differences between adult and aged rats in any case were found (Figure 2).

**Figure 3:**
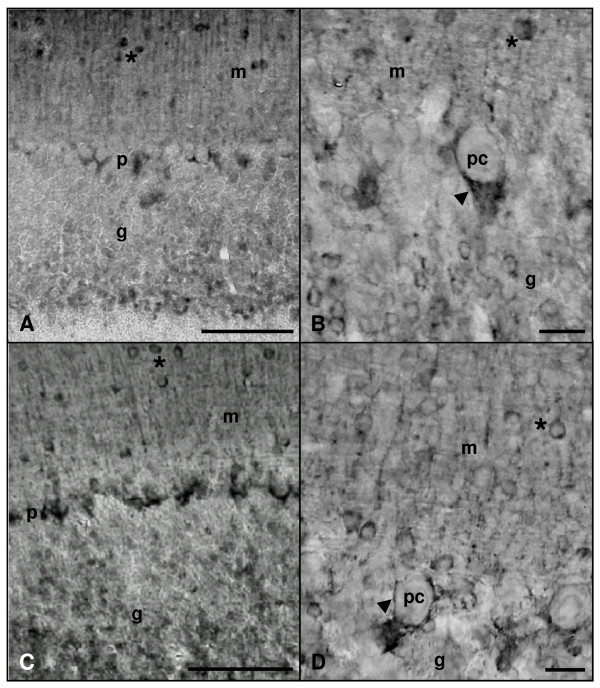
**Microphotographies of nNOS immunoreactivity in the cortex of rat cerebellum rostro-caudal sections**. Panels A and B: adult rat. Panels C and D: old rat. Immunopositive basket cells terminals (arrowheads) and stellate cells (asterisks) of the molecular layer (m) as well as granule cells in the granular layer (g) are immunolabelled in both groups (A-D). p: Purkinje cells layer; pc: Purkinje cells. Scale bars: A and C, 100 μm; B and D, 20 μm.

**Figure 4:**
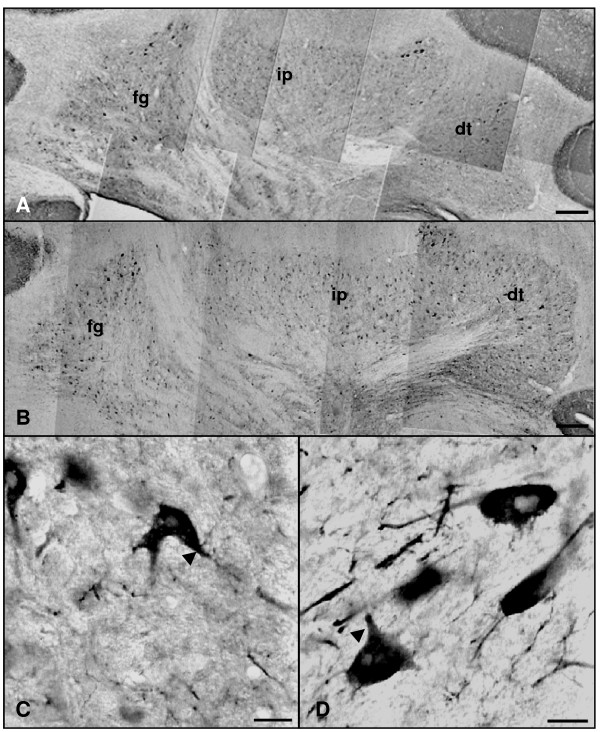
**Photomontage of nNOS immunoreactivity in the nuclei of rat cerebellum rostro-caudal sections**. Panels A and C: adult rat. Panels B and D old rat. nNOS immunoreactive neurons (arrowheads) in the fastigial (fg), interposed (ip), and dentate (dt) cerebellar nuclei of adult and aged rats are observed (A-D). Scale bars: A and B, 200 μm; C and D, 20 μm.

In addition, we performed an immunohistochemical study of the *in situ *expression and location of these three isoforms in the cerebellum of both groups. In the cerebellar cortex of adult and aged rats, nNOS-immunoreactive (nNOS-IR) basket-cell bodies as well as their processes surrounding Purkinje cell soma were detected (Figure 3). Some stellate cells of the molecular layer were also nNOS-IR in both experimental groups. On the other hand, granule cells were the most abundant nNOS-IR type detected in the cerebellum of both adult and aged animals. Neither marked Purkinje cell bodies nor processes were detected in any experimental group. nNOS-IR neurons of the deep cerebellar nuclei were noted in both age groups, where neuronal bodies and processes were stained in the three (fastigial, interposed and dentate) nuclei (Figure 4).

**Figure 5:**
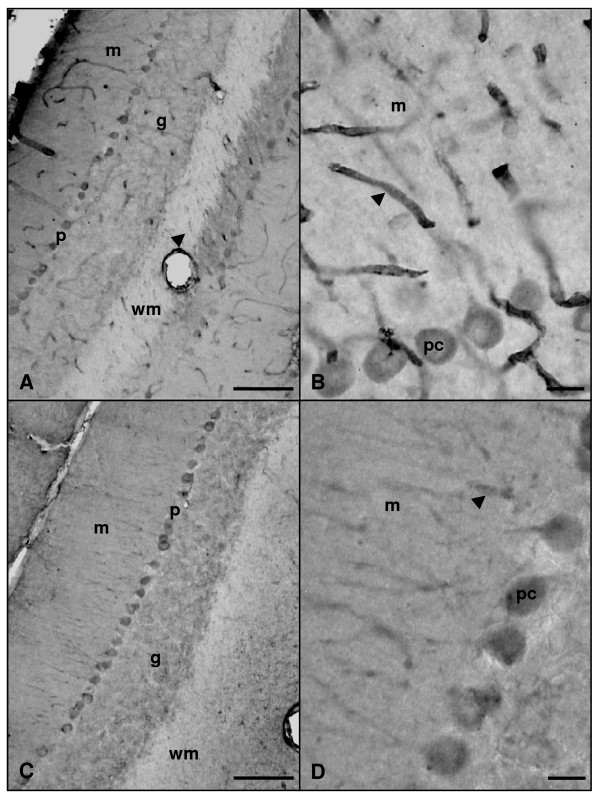
**Microphotographies of eNOS immunoreactivity in the cortex of rat cerebellum rostro-caudal sections**. Panels A and B: adult rat. Panels C and D: old rat. eNOS immunoreactive Purkinje cells (pc) and blood vessels (arrowheads) can be appreciated in both age groups. g: granular layer; m: molecular layer; p: Purkinje cells layer; wm: white matter. Scale bars: A and C, 100 μm; B and D, 20 μm.

**Figure 6:**
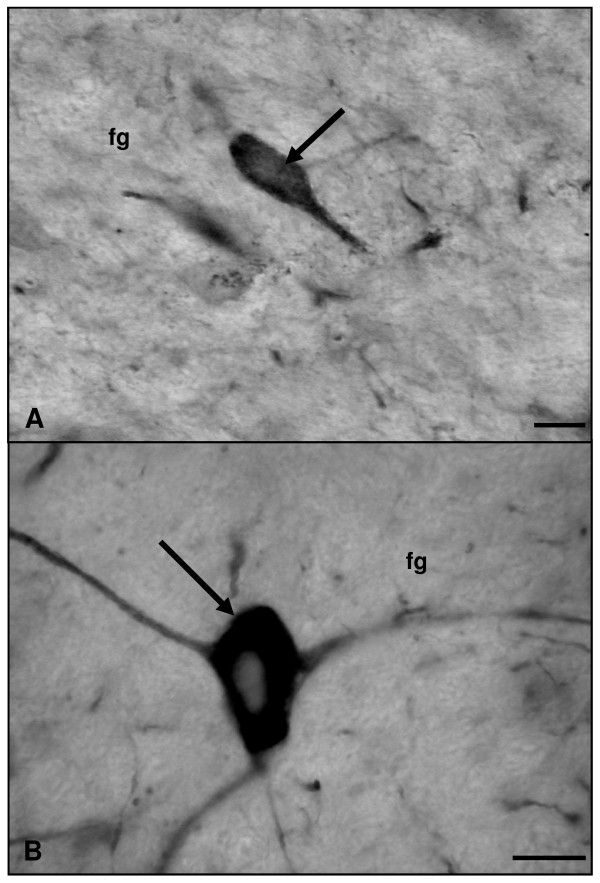
**Microphotographies of eNOS immunoreactivity in the deep nuclei of rat cerebellum rostro-caudal sections**. Panel A: adult rat. Panel B: old rat. Marked neurons (arrows) are observed in the fastigial nucleus (fg) of both adult and old rats (A, B). Scale bars: A and B, 20 μm.

Using a specific antibody against the endothelial NOS isoform, the only cerebellar eNOS-immunoreactive (eNOS-IR) cell populations detected in both experimental groups corresponded to vascular endothelial cells and Purkinje cells (Figure 5). Both in adult and in old animals, marked neurons of the three cerebellar nuclei were appreciated (Figure 6).

**Figure 7:**
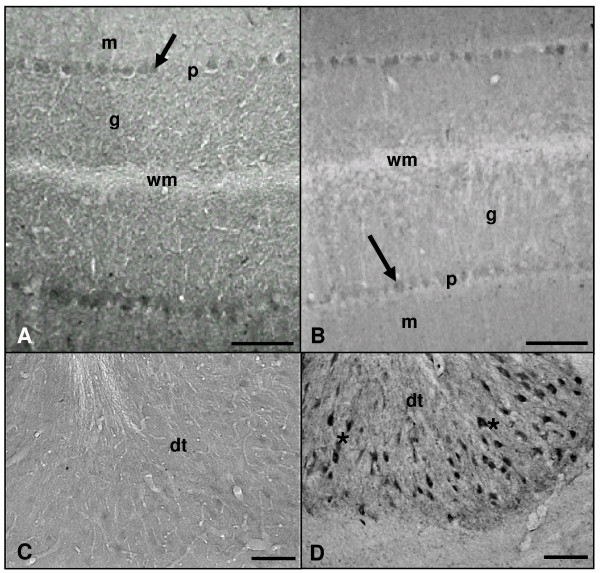
**Microphotographies of iNOS immunoreactivity in the cortex and nuclei of rat cerebellum rostro-caudal sections**. Panels A and C: adult rat. Panels B and D: old rat. Only Purkinje cells soma (arrows) seems to be positively marked in the cerebellar cortex when using an antibody against iNOS in both experimental groups (A, B). Marked neurons (asterisks) are observed in the dentate nucleus (dt) of old rats (D) but not in adult rats (C). g: granular layer; m: molecular layer; p: Purkinje cells layer; wm: white matter. Scale bars: A-D, 100 μm.

In relation to distribution of iNOS-immunoreactivity in the cerebellar cortex, only Purkinje cell bodies showed light but well-defined labelling in both experimental groups (Figure 7). On the other hand, cerebellar nuclei showed a different pattern of immunostaining with aging, since no reactive neurons were detected in adult rats but profusely immunostained neurons appeared in old rats (Figure 7).

### NADPH-diaphorase activity

**Figure 8:**
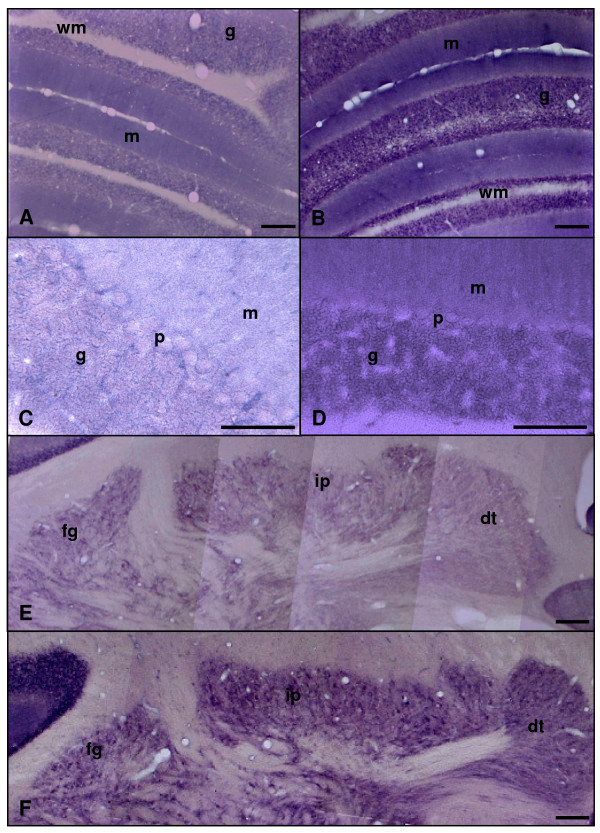
**Microphotographies of NADPH-diaphorase activity in the cortex and nuclei of rat cerebellum rostro-caudal sections**. Panels A, C and E: adult rat. Panels B, D and F: old rat. In adult and aged animals an uniform but diffuse mark is widely spread within the cerebellar cortex layers (A-D) and the three cerebellar nuclei: fastigial (fg), interposed (ip), and dentate (dt), although the intensity of the staining is higher in the aged animals (B, D). g: granular layer; m: molecular layer; p: Purkinje cells layer; wm: white matter. Scale bars: A, B, E and F, 200 μm; C and D, 100 μm.

In the molecular and granular layers of the cerebellar cortex, a diffuse and uniformly distributed NADPH-diaphorase histochemical staining was detected, this labelling being more intense in 26-month-old rats than in 4-month-old rats (Figure 8). Neurons of the deep cerebellar nuclei showed a diffuse but strong staining both in adult and aged rats, although the intensity was higher in old rats (Figure 8). Some blood vessels of the *arbor vitae *(i.e., the white matter surrounding the deep nuclei) were also labelled in both experimental groups (Figure 8).

### Nitrated protein expression and location

**Figure 9:**
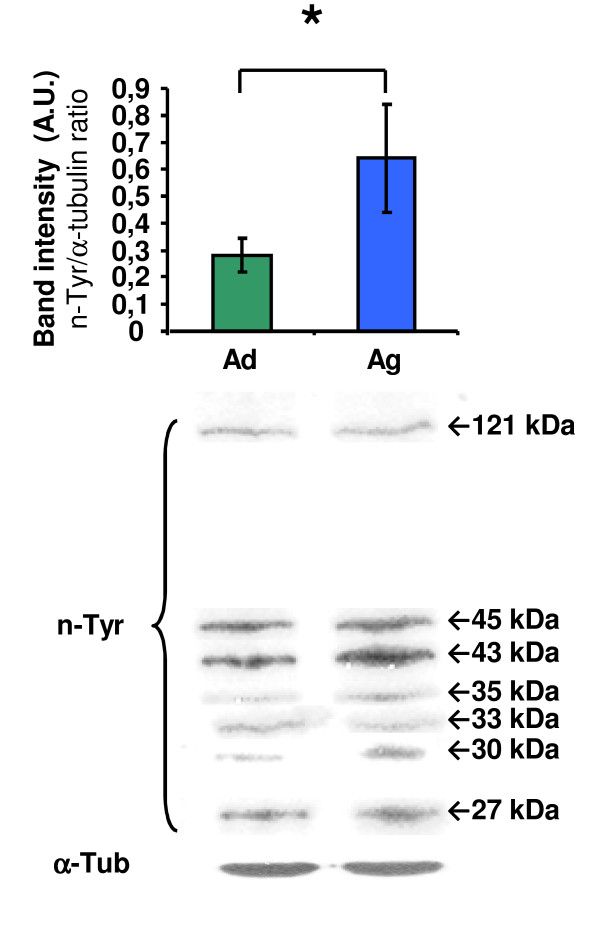
**Western-blot analysis of the nitrotyrosine modified proteins in the rat cerebellum**. Top panel: densitometric quantifications of bulk nitrotyrosine-modified protein expression in the cerebellum of adult and aged rats (Ad, Ag). Bottom panel: representative autoradiography of the different immunoreactive bands (121, 45, 43, 35, 33, 30, 27 kDa); α-tubulin was also included as a protein-loading control. Results are average values of 5 experimental animals in each group and are expressed as ratio of band intensities of nitrated proteins and its corresponding α-tubulin. Data are the means (± S.D.) of five determinations. A.U.: arbitrary units.* p < 0.05.

The western-blot analysis detected seven n-Tyr-immunoreactive (n-Tyr-IR) bands in the cerebellum of both age groups. These bands correspond to proteins with molecular weights of 121, 45, 43, 35, 33, 30 and 27 kDa. Notwithstanding, in the densitometric quantification, the total sum of nitrated proteins was considered, the bulk nitrated protein levels being significantly higher in 26-month-old rats (Figure 9).

**Figure 10:**
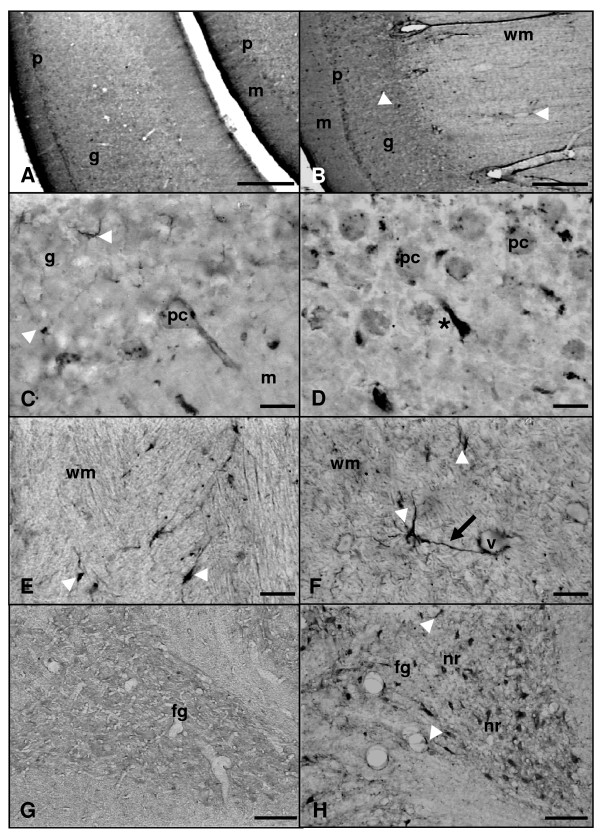
**Microphotographies of n-Tyr immunoreactivity in the cortex and nuclei of rat cerebellum rostro-caudal sections**. Panels A, E and G: adult rat. Panels B, C, D, F and H: old rat. n-Tyr immunoreactivity can be detected in the Purkinje cells (pc) of adult and old animals (A-D). In the aged animals, n-Tyr-IR glial cells (arrowheads) widely distributed within the white matter (wm) and the three layers of the cerebellar cortex are observed (B, C, F). Some glial cells (arrowheads) showing immunoreactive processes (arrow) surrounding blood vessels (v) were also present in old animals (F). In the adult individuals, n-Tyr-IR glial cells (arrowheads) are detected as well (A, E). Detail of retracted Purkinje cell (pc) apical processes (asterisk) in the aged rat cerebellum (D). Highly marked neurons (nr) and glial cells (arrowheads) are observed in the fastigial nucleus (fg) of old rats (H) but not in adult rats (G). g: granular layer; m: molecular layer; p: Purkinje cells layer. Scale bars: A and B, 200 μm; C-F, 20 μm; G and H, 100 μm.

In the cerebellar cortex of adult rats, nitrotyrosine immunoreactivity was restricted to Purkinje cells and to some glial cells located mainly in the white matter (Figure 100); no immunolabelling was observed in neurons of the other cortical layers (Figure 10). In the aged cerebellar cortex, n-Tyr-IR was found in glial cells as well, these cells being widely distributed within the cortex and white matter (Figure 10). Purkinje cells of the aged rats were also immunolabelled and, in a few cases, their upward processes rising towards the molecular layer were found to be retracted (Figure 10). In relation to cerebellar nuclei no immunoreaction was detected, either in neurons or glial cells of adult animals, although both types of cells were immunoreactive in aged rats (Figure 10).

### Total nitrogen oxides (NOx)

**Figure 11:**
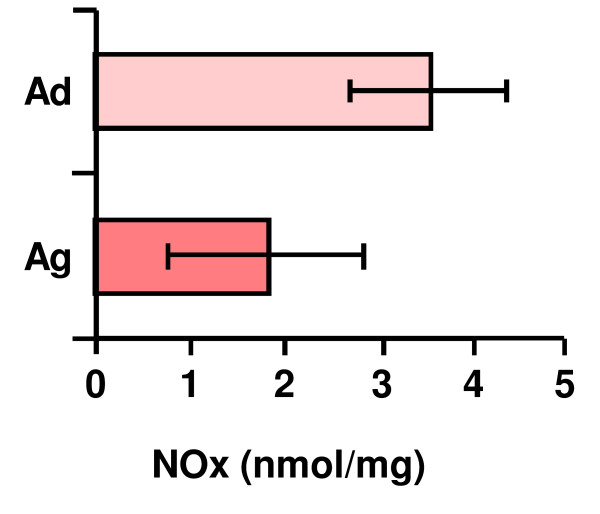
**Nitrate and nitrite levels in the rat cerebellum**. No statistical differences are detected when analysing the results from samples of adult (Ad) or aged (Ag) animals. Data are the means (± S.D.) of five determinations.

To ascertain age-related features of the NO production and accumulation in the cerebellum, tissue measurements of the NOx were made on denatured homogenates from adult (Ad) and aged (Ag) animals. Figure 11 shows the total nitrogen oxides (NOx) in the two experimental groups. Although there were higher NOx levels in adult rats, no statistical significance appeared between both ages.

## Discussion

In this study, we analyse the NO/NOS system and its involvement in the aging process in rat cerebellum. More specifically, the three NOS isoforms (nNOS, eNOS and iNOS) expression analysed by western-blot in the cerebellum of adult and aged individuals did not show any change with aging. This result confirms previous studies of our group [4], which only found changes in the expression of these enzymes when comparing young rats (sexually immature) with adult and old ones, but not between these two last groups. However, our results regarding NOS cellular distribution and activity, show new data that make it possible to appreciate age-related changes not detected by means of protein-expression studies solely.

Regarding the location of the three NOS isoforms within the different cell populations of the cerebellum, our data on adult animals were generally consistent with those previously reported by other authors [19-23]. In older animals, however, we found some changes related mostly to iNOS. These age-dependent changes suggest the possibility that the NO pathways involved in the cerebellar functions, previously established by other authors [24-26], might suffer certain changes during senescence. Although further analyses will be necessary to elucidate the positive/negative role of such changes, previous studies of our group [27] have suggested that aging appears to induce a type of preconditioning towards ischemic hypoxia in the cerebellum, possibly due to high baseline nitration levels, a feature also found in this study as we discuss later.

In relation to the immunohistochemical results, we have observed that the basket-cell processes surrounding Purkinje cell soma were nNOS-positive; this suggests that Purkinje cells may act as NO targets, a fact consistent both with the presence of nitrated proteins in Purkinje cells. These findings regarding nNOS location coincide with those described in adult rats [20,22], and also indicate that there are no significant changes associated to aging in relation to the nitrergic system involved in Purkinje cells functions.

As previously mentioned, eNOS expression in the cerebellum shows no age-related changes when analysed by means of western-blot. This result coincides with those found by other authors, both in cerebral cortex [28] and cerebellum [4,18]. Few works show eNOS location in the cerebellum; all these studies agree on the eNOS vascular location [22]; in addition, studies by our group [29] and others [30], have also found this isoform in Purkinje cells and neurons of the cerebellar nuclei as well.

According to our data, iNOS immunoreactivity in the cerebellar cortex is restricted to a faint mark in Purkinje cells in 4 and 26-month-old rats. On the contrary, while in adult individuals no iNOS-immunoreactive (iNOS-IR) neurons were observed in the cerebellar nuclei, interestingly, in the aged rats intensely marked neurons appeared. However, although cerebellar iNOS location pattern differs between both age groups, the overall iNOS expression in the cerebellum does not show significant differences with aging. In this sense, previous studies of our group [4] and other authors [31] have shown no changes in the expression of iNOS during aging as well, but only as a result of sexual maturation.

After treatment with aldehydes, only NOS-related NADPH-diaphorase (NADPH-d) activity remains, so that NADPH-d histochemical staining is usually used as a complementary method for the indirect demonstration of NOS activity by light microscopy [32,33]. Further, some authors [34] have demonstrated a patchy expression pattern in the cerebellum of adult mice, but only in the granular layer. However, in the cerebellum of both adult and old rats, we detected a diffuse and uniformly distributed NADPH-diaphorase histochemical staining. Thus, we found similar location patterns between the two experimental groups, although the intensity of the histochemical mark is higher in the 26-month-old rat group. In addition, some studies have demonstrated an association of NADPH-d activity with NOS activity [35-38]. Considering all these facts, we could postulate that although overall NOS expression does not vary with age, NOS activity or at least that of one of its isoforms seems to be increased in older animals.

Our results also indicate an increase in protein nitration with aging, and the presence of n-Tyr-immunoreactive (n-Tyr-IR) Purkinje cells in both age groups. These data are consistent with those reported by Chung and collaborators [5]. In addition, in the aged cerebellum we have found some Purkinje cells that show retraction phenomena affecting its apical processes, which could in turn affect the functionality of the cerebellar cortex. This phenomenon has been previously described in the ischemic rat cerebellum by Rodrigo and collaborators [20]. We have also detected n-Tyr-IR glial cells, which could imply a mismatch in their defence and trophic support functions, thus influencing cerebellar neurons life expectancy as previously described by other studies [39] during aging.

The NOx levels analysed in this study do not vary significantly with the age of the rat; this fact is consistent with the stability in NOS expression, but not with the increased diaphorase activity previously described. Nevertheless, If we consider the protein nitration boost detected during aging, it could be interpreted that NO produced during senescence is assigned to a greater extent in the aged animals to protein nitration. The experimental procedure used for NOx determination detects only NO measured as nitrate plus nitrite, but not the NO bound to tyrosine residues. This fact could explain the gap between the increased protein nitration data and the invariability in NOx levels detected during aging. Moreover, Jesko and collaborators [40] stated that nNOS is the main responsible, by means of phosphorylation, for NOS activity during aging. NADPH-d activity can also be related to eNOS [35] as well, but some studies indicate a decline in the catalytic activity of eNOS in the aged cerebellum [18]. Taking into account these data and that we have not found any differences between nNOS and eNOS expression or location with aging, we could hypothesise that the increased NADPH-d activity, and the subsequent increase in protein nitration detected during aging may not be due to eNOS, but further analysis are required.

## Conclusions

In summary, the NO/NOS system suffers from a number of changes with aging in the cerebellum: 1) A change in the cellular distribution of iNOS but not in its expression. 2) Greater NADPH-d activity, and 3) An increased protein nitration. These results suggest that there are changes affecting the NO/NOS system and the destiny of the NO produced during aging in the cerebellum, such as variations in the production of reactive oxygen or decreases in anti-oxidation protection mechanisms.

## Methods

### Animals

The study was performed on 10 adult (Ad) (4-5 months old) and 10 aged (Ag) (26-27 months old) male albino Wistar rats kept under standard conditions of light and temperature and allowed ad *libitum *access to food and water. All procedures were carried out in accordance with the European Communities Council Directive (86/609/EEC), reviewed by the Ethics Committee of the Spanish Council for Scientific Research and approved by the Committee of Bioethics of the University of Jaén.

### Antibodies

All the antibodies used in this study have been previously tested by our group and others, showing specificity for their respective antigens [4,27,29,41-43].

### Western-blot analysis

For western-blot analysis, the cerebella from 5 animals from each group were dissected and stored at -80°C until used. Tissues were homogenized in 1/3 (w/v) of 30 mM Tris-HCl, pH 7.4 containing 0.5 mM DTT, 1 mM EDTA, 1% SDS and protease inhibitors. The resulting homogenates were centrifuged for 1 h at 100,000xg. All the procedures were performed at 0 - 4°C. Protein concentrations in the supernatants were determined by the Bradford method [44]. Equal amounts of the denatured proteins per lane were loaded and separated on a 7.5% (NOS) and 10% (Nitrotyrosine) SDS-polyacrylamide gel (Mini Protean 3. Bio-Rad, Hercules, CA, EE.UU.), as described by Laemmli [45]. Afterwards, proteins were transferred to a PVDF membrane (Immobilon-P. Ref.: IPVH00010. Millipore, Billerica, MA, USA). The membranes were blocked with 5% powdered non-fat milk in 25 mM Tris-HCl, pH 7.6, 137 mM NaCl, 2.6 mM KCl, 0.1% Tween-20 and incubated overnight at 4°C with diluted, rabbit polyclonal anti-nNOS (1:3000, gift from V. Riveros-Moreno of Welcome Research Laboratories, Berkenhem, UK), monoclonal anti-iNOS and anti-eNOS (1:800, Transduction, Lexington, KY, USA), and rabbit polyclonal anti-nitrotyrosine (1:2000, produced by our group) [46] in blocking solution. Bound antibody was revealed by means of an enhanced chemiluminescence kit (Amersham, Little Chalfont, UK) according to the manufacturer's instructions. After immunodetection, membranes were probed with anti-α-tubulin (Sigma, St. Louis, MO, USA) as a loading control. The relative amount of the proteins in each sample was quantified by densitometric scanning and expressed as arbitrary units (A.U.).

### Immunohistochemical procedure

For immunohistochemical procedures, cerebella from 5 adult (Ad) and 5 aged (Ag) rats were extracted and processed as follows. Deeply anaesthetized animals (15 mg/100 g B.W. i.p.; Ketolar. Parke Davis, Madrid, Spain) were perfused through the left ventricle with 50 ml of carbogenated 0.01 M phosphate-buffered saline (PBS; pH 7.4), and then with 300 ml of 4% paraformaldehyde in 0.1 M phosphate buffer (PB). The cerebella were removed and then postfixed for a further 4 h in the same fixative at room temperature. Samples were then cryoprotected by immersion overnight at 4°C in 0.1 M PB containing 30% sucrose. After that, the cerebella were embedded in O.C.T medium and frozen in 2-methylbutane pre-chilled in liquid nitrogen. Serial rostro-caudal sections (40 μm) were cut using a cryostat (Cryocut 1800. Reichert Jung, Wetzlar, Germany). Free-floating sections were incubated for 4 h in PBS containing 0.1% Triton X-100, and then in: nNOS, 1:900 (this antiserum was a gift from Dr. V. Riveros Moreno of Welcome Research Laboratories, Beckenham, UK; [43]); eNOS and iNOS, 1:150 (Transduction, Lexington, KY, USA); or in nitrotyrosine, 1:1,000 [46] antisera diluted in PBS containing 0.2% Triton X-100, overnight at 4°C. After several rinses in PBS, and depending on the origin of the primary antisera, the sections were incubated with biotinylated goat anti-rabbit IgG or biotinylated rabbit anti-mouse IgG, 1:100 (Pierce, Rockford, IL, USA), and processed by the avidin-biotin peroxidase complex (ABC) procedure (Pierce, Rockford, IL, USA). The peroxidase activity was demonstrated following the nickel-enhanced diaminobenzidine assay [47]. Control procedures were carried out on adjacent sections of the same tissues. No immunolabelling was detected when the primary antibody was either omitted or replaced with an equivalent concentration of pre-immune serum. Sections were then mounted on slides, dehydrated, and covered using DPX (Fluka, Madrid, Spain).

### Histochemistry

Free-floating sections were incubated for 4 h in PBS containing 0.1% Triton X-100. After several washes in 0.1 M Tris-HCl, pH 7.4 buffer, they were incubated in the dark, for 45 min at 37°C, in 0.1 M Tris-HCl, pH 7.4, containing 1 mM β-NADPH and 2 mM NBT (in 70% dimethylformamide). The sections were then washed twice with 0.1 M Tris-HCl, pH 7.4, quickly dehydrated in a graded ethanol series, cleared and mounted in DPX (Fluka, Madrid, Spain).

### NO production

NO production was determined by tissue accumulation of nitrite and nitrate in the cerebellum of the same animals used for western-blot analysis. Briefly, a portion of tissue specimens from 5 animals of each group was homogenized in 3 volumes (w/v) of PBS (pH 7.6) at 4°C. Homogenates were then sonicated and centrifuged at 100,000xg for 60 min at 4°C. The nitrate plus nitrite (NOx) was determined in the supernatants using a colorimetric kit according to the manufacturer's instructions (Nitrate/Nitrite colorimetric Assay Kit. Cayman Chemical, Ann Arbor, MI, USA).

### Statistical analysis

Data are expressed as means ± S.D. Student's t test was performed to evaluate the significance of differences between groups, accepting p < 0.05 as the level of significance.

## List of abbreviations

(ad): adult; (ag): aged; (eNOS): endothelial nitric oxide synthase; (IR): immunoreactivity; (iNOS): inducible nitric oxide synthase; (nNOS): neuronal nitric oxide synthase; (NO): nitric oxide; (NOS): nitric oxide synthase; (NOx): nitrogen oxides; (n-Tyr): nitrotyrosine; (PB): phosphate buffer.

## Authors' contributions

SB carried out all the experiments, participated in the data analysis and drafted the manuscript. FJM carried out the immunohistochemical and histochemical experiments. LC participated in the western blotting and data analysis. MLM participated in the design of the study and performed the statistical analysis. RH helped with the NOx studies. AJ helped with the immunohistochemical and histochemical experiments. AR participated in the western blotting analysis. EML helped with the western blotting data analysis. ES participated in NOx studies data analysis. MAP conceived of the study, and participated in its design and coordination and helped to draft the manuscript. All authors read and approved the final manuscript.

## References

[B1] Forstermann U Gorsky LD Pollock JS Schmidt HH Heller M Murad F Regional distribution of EDRF/NO-synthesizing enzyme(s) in rat brain Biochem Biophys Res Commun 1990 168 727 732 10.1016/0006-291X(90)92382-A 1692215

[B2] Peinado MA del Moral ML Esteban FJ Martinez-Lara E Siles E Jimenez A Hernandez-Cobo R Blanco S Rodrigo J Pedrosa JA [Aging and neurodegeneration: molecular and cellular bases] Rev Neurol 2000 31 1054 1065 11190874

[B3] Martinez-Romero R Canuelo A Martinez-Lara E Hernandez R Del Moral ML Pedrosa JA Peinado MA Siles E Aging affects but does not eliminate the enzymatic antioxidative response to hypoxia/reoxygenation in cerebral cortex Exp Gerontol 2006 41 25 31 10.1016/j.exger.2005.09.009 16260109

[B4] Siles E Martinez-Lara E Canuelo A Sanchez M Hernandez R Lopez-Ramos JC Del Moral ML Esteban FJ Blanco S Pedrosa JA Age-related changes of the nitric oxide system in the rat brain Brain Res 2002 956 385 392 10.1016/S0006-8993(02)03575-8 12445710

[B5] Chung YH Shin CM Joo KM Kim MJ Cha CI Immunohistochemical study on the distribution of nitrotyrosine and neuronal nitric oxide synthase in aged rat cerebellum Brain Res 2002 951 316 321 10.1016/S0006-8993(02)03261-4 12270511

[B6] Harman D Free radical theory of aging: effect of free radical reaction inhibitors on the mortality rate of male LAF mice J Gerontol 1968 23 476 482 5723482 10.1093/geronj/23.4.476

[B7] McCann SM The nitric oxide hypothesis of brain aging Exp Gerontol 1997 32 431 440 10.1016/S0531-5565(96)00154-4 9315447

[B8] McCann SM Licinio J Wong ML Yu WH Karanth S Rettorri V The nitric oxide hypothesis of aging Exp Gerontol 1998 33 813 826 10.1016/S0531-5565(98)00050-3 9951625

[B9] Daniel H Hemart N Jaillard D Crepel F Long-term depression requires nitric oxide and guanosine 3':5' cyclic monophosphate production in rat cerebellar Purkinje cells Eur J Neurosci 1993 5 1079 1082 10.1111/j.1460-9568.1993.tb00961.x 7506617

[B10] Hartell NA cGMP acts within cerebellar Purkinje cells to produce long term depression via mechanisms involving PKC and PKG Neuroreport 1994 5 833 836 10.1097/00001756-199403000-00024 7517198

[B11] Lev-Ram V Makings LR Keitz PF Kao JP Tsien RY Long-term depression in cerebellar Purkinje neurons results from coincidence of nitric oxide and depolarization-induced Ca2+ transients Neuron 1995 15 407 415 10.1016/0896-6273(95)90044-6 7646893

[B12] Beckman JS Koppenol WH Nitric oxide, superoxide, and peroxynitrite: the good, the bad, and ugly Am J Physiol 1996 271 C1424 1437 8944624 10.1152/ajpcell.1996.271.5.C1424

[B13] Eliasson MJ Huang Z Ferrante RJ Sasamata M Molliver ME Snyder SH Moskowitz MA Neuronal nitric oxide synthase activation and peroxynitrite formation in ischemic stroke linked to neural damage J Neurosci 1999 19 5910 5918 10407030 10.1523/JNEUROSCI.19-14-05910.1999 PMC6783066

[B14] Hanafy KA Krumenacker JS Murad F NO, nitrotyrosine, and cyclic GMP in signal transduction Med Sci Monit 2001 7 801 819 11433215

[B15] Ischiropoulos H Beckman JS Oxidative stress and nitration in neurodegeneration: cause, effect, or association? J Clin Invest 2003 111 163 169 12531868 10.1172/JCI17638 PMC151889

[B16] Crepel F Jaillard D Protein kinases, nitric oxide and long-term depression of synapses in the cerebellum Neuroreport 1990 1 133 136 10.1097/00001756-199010000-00013 2129866

[B17] Shimizu-Albergine M Rybalkin SD Rybalkina IG Feil R Wolfsgruber W Hofmann F Beavo JA Individual cerebellar Purkinje cells express different cGMP phosphodiesterases (PDEs): in vivo phosphorylation of cGMP-specific PDE (PDE5) as an indicator of cGMP-dependent protein kinase (PKG) activation J Neurosci 2003 23 6452 6459 12878685 10.1523/JNEUROSCI.23-16-06452.2003 PMC6740622

[B18] Strosznajder JB Jesko H Zambrzycka A Eckert A Chalimoniuk M Age-related alteration of activity and gene expression of endothelial nitric oxide synthase in different parts of the brain in rats Neurosci Lett 2004 370 175 179 10.1016/j.neulet.2004.08.013 15488318

[B19] Leon-Chavez BA Aguilar-Alonso P Gonzalez-Barrios JA Eguibar JR Ugarte A Brambila E Ruiz-Arguelles A Martinez-Fong D Increased nitric oxide levels and nitric oxide synthase isoform expression in the cerebellum of the taiep rat during its severe demyelination stage Brain Res 2006 1121 221 230 10.1016/j.brainres.2006.08.097 17022950

[B20] Rodrigo J Alonso D Fernandez AP Serrano J Richart A Lopez JC Santacana M Martinez-Murillo R Bentura ML Ghiglione M Uttenthal LO Neuronal and inducible nitric oxide synthase expression and protein nitration in rat cerebellum after oxygen and glucose deprivation Brain Res 2001 909 20 45 10.1016/S0006-8993(01)02613-0 11478918

[B21] Rodrigo J Springall DR Uttenthal O Bentura ML Abadia-Molina F Riveros-Moreno V Martinez-Murillo R Polak JM Moncada S Localization of nitric oxide synthase in the adult rat brain Philos Trans R Soc Lond B Biol Sci 1994 345 175 221 10.1098/rstb.1994.0096 7526408

[B22] Serrano J Encinas JM Salas E Fernandez AP Castro-Blanco S Fernandez-Vizarra P Bentura ML Rodrigo J Hypobaric hypoxia modifies constitutive nitric oxide synthase activity and protein nitration in the rat cerebellum Brain Res 2003 976 109 119 10.1016/S0006-8993(03)02691-X 12763628

[B23] Southam E Morris R Garthwaite J Sources and targets of nitric oxide in rat cerebellum Neurosci Lett 1992 137 241 244 10.1016/0304-3940(92)90413-2 1316590

[B24] Hartell NA Receptors, second messengers and protein kinases required for heterosynaptic cerebellar long-term depression Neuropharmacology 2001 40 148 161 10.1016/S0028-3908(00)00107-6 11077081

[B25] Reynolds T Hartell NA Roles for nitric oxide and arachidonic acid in the induction of heterosynaptic cerebellar LTD Neuroreport 2001 12 133 136 10.1097/00001756-200101220-00034 11201073

[B26] Schweighofer N Ferriol G Diffusion of nitric oxide can facilitate cerebellar learning: A simulation study Proc Natl Acad Sci USA 2000 97 10661 10665 10.1073/pnas.97.19.10661 10984547 PMC27081

[B27] Blanco S Castro L Hernandez R Del Moral ML Pedrosa JA Martinez-Lara E Siles E Peinado MA Age modulates the nitric oxide system response in the ischemic cerebellum Brain Res 2007 1157 66 73 10.1016/j.brainres.2007.01.141 17544383

[B28] Liu P Smith PF Appleton I Darlington CL Bilkey DK Age-related changes in nitric oxide synthase and arginase in the rat prefrontal cortex Neurobiol Aging 2004 25 547 552 10.1016/j.neurobiolaging.2003.07.003 15013576

[B29] Hernandez R Martinez-Lara E Del Moral ML Blanco S Canuelo A Siles E Esteban FJ Pedrosa JA Peinado MA Upregulation of endothelial nitric oxide synthase maintains nitric oxide production in the cerebellum of thioacetamide cirrhotic rats Neuroscience 2004 126 879 887 10.1016/j.neuroscience.2004.04.010 15207323

[B30] Shin T Weinstock D Castro MD Hamir AN Wampler T Walter M Kim HY Acland H Immunohistochemical localization of endothelial and inducible nitric oxide synthase within neurons of cattle with rabies J Vet Med Sci 2004 66 539 541 10.1292/jvms.66.539 15187365

[B31] Vernet D Bonavera JJ Swerdloff RS Gonzalez-Cadavid NF Wang C Spontaneous expression of inducible nitric oxide synthase in the hypothalamus and other brain regions of aging rats Endocrinology 1998 139 3254 3261 10.1210/en.139.7.3254 9645701

[B32] Kugler P Hofer D Mayer B Drenckhahn D Nitric oxide synthase and NADP-linked glucose-6-phosphate dehydrogenase are co-localized in brush cells of rat stomach and pancreas J Histochem Cytochem 1994 42 1317 1321 7523487 10.1177/42.10.7523487

[B33] Roufail E Stringer M Rees S Nitric oxide synthase immunoreactivity and NADPH diaphorase staining are co-localised in neurons closely associated with the vasculature in rat and human retina Brain Res 1995 684 36 46 10.1016/0006-8993(95)00394-6 7583202

[B34] Schilling K Schmidt HH Baader SL Nitric oxide synthase expression reveals compartments of cerebellar granule cells and suggests a role for mossy fibers in their development Neuroscience 1994 59 893 903 10.1016/0306-4522(94)90293-3 7520135

[B35] Andronowska A Wasowska B Calka J Doboszynska T Localization and correlation between NADPH-diaphorase and nitric oxide synthase isoforms in the porcine uterus during the estrous cycle Cell Tissue Res 2005 321 243 250 10.1007/s00441-005-1085-9 15951992

[B36] Necchi D Virgili M Monti B Contestabile A Scherini E Regional alterations of the NO/NOS system in the aging brain: a biochemical, histochemical and immunochemical study in the rat Brain Res 2002 933 31 41 10.1016/S0006-8993(02)02302-8 11929633

[B37] Paakkari I Lindsberg P Nitric oxide in the central nervous system Ann Med 1995 27 369 377 10.3109/07853899509002590 7546627

[B38] Page GK Morton AJ Correlation of neuronal loss with increased expression of NADPH diaphorase in cultured rat cerebellum and cerebral cortex Brain Res 1995 697 157 168 10.1016/0006-8993(95)00801-V 8593572

[B39] Sloane JA Hollander W Moss MB Rosene DL Abraham CR Increased microglial activation and protein nitration in white matter of the aging monkey Neurobiol Aging 1999 20 395 405 10.1016/S0197-4580(99)00066-4 10604432

[B40] Jesko H Chalimoniuk M Strosznajder JB Activation of constitutive nitric oxide synthase(s) and absence of inducible isoform in aged rat brain Neurochem Int 2003 42 315 322 10.1016/S0197-0186(02)00098-0 12470705

[B41] Del Moral ML Esteban FJ Hernandez R Blanco S Molina FJ Martinez-Lara E Siles E Viedma G Ruiz A Pedrosa JA Peinado MA Immunohistochemistry of neuronal nitric oxide synthase and protein nitration in the striatum of the aged rat Microsc Res Tech 2004 64 304 311 10.1002/jemt.20081 15481048

[B42] Martinez-Lara E Canuelo AR Siles E Hernandez R Del Moral ML Blanco S Pedrosa JA Rodrigo J Peinado MA Constitutive nitric oxide synthases are responsible for the nitric oxide production in the ischemic aged cerebral cortex Brain Res 2005 1054 88 94 10.1016/j.brainres.2005.06.060 16054596

[B43] Riveros-Moreno V Heffernan B Torres B Chubb A Charles I Moncada S Purification to homogeneity and characterisation of rat brain recombinant nitric oxide synthase Eur J Biochem 1995 230 52 57 10.1111/j.1432-1033.1995.tb20533.x 7541350

[B44] Bradford MM A rapid and sensitive method for the quantitation of microgram quantities of protein utilizing the principle of protein-dye binding Anal Biochem 1976 72 248 254 10.1016/0003-2697(76)90527-3 942051

[B45] Laemmli UK Cleavage of structural proteins during the assembly of the head of bacteriophage T4 Nature 1970 227 680 685 10.1038/227680a0 5432063

[B46] Uttenthal LO Alonso D Fernandez AP Campbell RO Moro MA Leza JC Lizasoain I Esteban FJ Barroso JB Valderrama R Neuronal and inducible nitric oxide synthase and nitrotyrosine immunoreactivities in the cerebral cortex of the aging rat Microsc Res Tech 1998 43 75 88 10.1002/(SICI)1097-0029(19981001)43:1<75::AID-JEMT11>3.0.CO;2-0 9829462

[B47] Shu SY Ju G Fan LZ The glucose oxidase-DAB-nickel method in peroxidase histochemistry of the nervous system Neurosci Lett 1988 85 169 171 10.1016/0304-3940(88)90346-1 3374833

